# Rendering Rubber Gas Tubing Odorless

**Published:** 1884-06

**Authors:** 


### Rendering Rubber Gas Tubing Odorless.

The Mohrisch-Schleisher *Gewerbhalle* recommends for deodorizing rubber gas tubing, that equal volumes of 36 per cent. alcohol and good linseed oil be shaken together to form a homogeneous mixture, and the moderately-stretched tube rubbed with a small rag, on which has been dropped a little of the mixure, until quite dry. The operation is to be repeated three or four times at intervals of a few days. The flexibility or color of the tube is said not to be impaired, while the rubber is rendered gas tight.

